# A Noble Dog

**Published:** 1887-04-30

**Authors:** F. O. Morris

**Affiliations:** Rector of Nunburnholme, Yorkshire; author of "A History of British Birds," etc.


### Incidents of Animal Life.

Collated by the Rev. F. O. Morris, B.A.,
Rector of Nunburnholme, Yorkshire; author of "A History of British Birds," etc.

### A NOBLE DOG.

Mr. Edward A. Whiston, of Boston, U.S.A., sends us the following true story about a dog, for the accuracy of which he vouches, the facts being strictly as stated:—

The story I am going to tell you is about a dog we had when I was a child. He was a thorough-bred English bull-dog, pure white, with the exception of one black spot on his forehead. A large, noble-looking animal, but fierce, and of such strength that strangers much preferred to see him strongly chained to his kennel than at large. When young, he belonged to an officer in the British army, and had travelled with his master from England to India, and from there to Canada. After a short stay there, the regiment the officer belonged to was recalled to England, and Leo, for that was the dog's name, was left as a parting gift to my father. The dog mourned the loss of his old master a long time, but finally became reconciled to his new home. He was a great rover, and used to travel the country round for many miles, hunting up all the big dogs, and challenging them to fight. I need hardly say that very few had courage to tackle Leo. He took no notice whatever of small dogs. From this habit of his we got into the way of calling him Ranger, and this gradually became his name.

We were at breakfast the morning when the incident I am going to relate happened. Ranger lay on a mat near the door of the room, with his head between his paws, resting, but wide awake to everything that was going on. A great, sleek cat, the pet of one of my younger sisters, was stretched on a chair near the breakfast-table, apparently unconscious of everything but the pleasant spring sunshine that streamed into the room through the large bay-window; and flitting here and there, picking up the crumbs scattered by my father for it, was a beautiful, yellow canary. Suddenly this peaceful scene was broken by the spring of both cat and dog towards the corner of the table where the bird was. We next saw pussy fly out of the room, like an arrow shot from a bow, and Ranger stand motionless at my father's side, his mouth firmly closed, but with a pleased expression on his face. The canary was nowhere to be seen. The whole thing was so sudden, that it was hard to realise what had taken place. We certainly thought Ranger had been quicker than the cat, and had got our dear little bird for his breakfast.

You can imagine how we all mourned the loss of our pet, and how reproachfully my father looked down at the dog, saying, "Oh, Ranger, Ranger!" The noble fellow seemed to know he was misunderstood, for with one glance to see that the cat was really gone, he opened his mouth. What was our surprise and joy to see fly out of it, unhurt, though frightened, our little songster, who, after a little dressing of his ruffled feathers, was soon hopping around the room again.

Was not the mouth of that fierce bull-dog a strange ark of safety for a tiny canary when threatened by its wily enemy, the cat, and how wonderfully the dog did the only thing he had time to do to save the little bird's life! Ranger was so incensed at the cat's conduct on this occasion, that he kept her for three days a prisoner on the eaves of the barn; and after that time pussy had to steal into the house unobserved, until the dog's anger had time to cool.

